# Species-specific surface dwell times and accumulation of microbes investigated by holographic microscopy

**DOI:** 10.1098/rsta.2024.0265

**Published:** 2025-09-11

**Authors:** Emma E. Brock, Samuel A. Matthews, Eli J. Cohen, Shuichi Nakamura, Laurence G. Wilson

**Affiliations:** ^1^ Yusuf Hamied Department of Chemistry, University of Cambridge, Cambridge, UK; ^2^ School of Physics, Engineering & Technology, University of York, York, UK; ^3^ Department of Life Sciences, Imperial College London, London, UK; ^4^ Department of Applied Physics, Tohoku University, Sendai, Miyagi Prefecture, Japan

**Keywords:** holographic microscopy, microswimmers, bacterial motility, active matter

## Abstract

Microscopic swimmers, such as bacteria and archaea, are paradigmatic examples of active matter systems. The study of these systems has given rise to novel concepts such as rectification of bacterial swimmers, in which microstructures can passively separate swimmers from non-swimming, inert particles. Many bacteria and archaea swim using rotary molecular motors to drive helical propellers called flagella or archaella. The arrangement of these filaments around the cell body varies between species, yielding a range of swimming patterns. Solid boundaries might affect these swimming patterns in different ways, limiting the generality of artificial rectification devices. We performed three-dimensional cell tracking on four species of bacteria with different flagellation patterns (*Escherichia coli*, *Salmonella enterica*, *Campylobacter jejuni* and *Shewanella putrefaciens*), and compared these to two environmental isolates of archaea, previously identified with the genera *Haloarcula* and *Haloferax*. Despite differences in the cells’ flagellation patterns, swimming speeds and swimming patterns, we find that the shapes of cell tracks as individuals leave surfaces are surprisingly consistent. This information reduces the potential parameter space for the design of artificial rectification devices, and increases the possibility of constructing a multi-species bacterial rectifier.

This article is part of the theme issue ‘Biological fluid dynamics: emerging directions’.

## 1. Introduction

Microorganisms use their ability to move (their motility) to explore their environments and optimize their chances of survival. Set against the metabolic cost of maintaining motility apparatus, this suggests that motility conveys a strong selective advantage. The ability to move is usually coupled to the ability to sense the environment. Cells swim towards nutrients or away from detrimental substances. Navigation on the microscale is affected by the randomizing effects of Brownian motion, however. Micrometre-scale objects such as bacteria experience fluctuations in their position, but also in their orientation. The latter prevent a bacterium swimming in a straight line, and necessitate frequent changes of direction in response to temporal cues [1]. The canonical example is that of *Escherichia coli*, which executes straight ‘runs’ of approximately 1 s punctuated by ‘tumbles’ lasting approximately 0.1 s. At a large scale, this run/tumble motif results in a random walk with an effective diffusion coefficient that can be calculated analytically from details of the swimming patterns [2].

Swimming bacteria and archaea are propelled by flagellar (or archaellar) systems of helical filaments driven by rotary motors. Strikingly, these adaptations to swimming in a low-Reynolds-number environment arrived at the same operating principle by two different evolutionary routes, from distinct progenitor structures and using different energy sources [3–6]. Many bacterial and archaeal cell bodies are broadly spherocylindrical, but the flagellar arrangement varies considerably between species.

Some have a single, polar flagellum (*Pseudomonas aeruginosa, Vibrio cholerae*), whereas others may have a flagellum at each pole (such as the amphitrichously flagellated *Campylobacter jejuni*) or several distributed about the cell surface (peritrichously flagellated *E. coli* and *Salmonella enterica*). The distributions of archaella around archaeal cell bodies are less well studied, though bundles of archaeal flagella have been revealed in electron micrographs [7]. The layout of flagella/archaella around the cell body governs how they reorient: peritrichous cells typically exhibit a run/tumble random walk with a broad range of angles between runs [8], whereas cells with a polar flagellum or flagellar bundle undergo reversals or exploit a flagellar buckling transition for ‘run–reverse–flick’ motif [9,10]. These motility patterns (and others) are observed when cells swim in bulk fluid; the presence of solid interfaces can disrupt these patterns and lead to novel behaviour.

Interactions with surfaces are central to the life cycles of many microbes. When a freely swimming bacterial or archaeal cell encounters a solid boundary, one of several outcomes may result. The cells can transiently or permanently attach to surfaces, and may then form biofilms [11,12]. After attachment, they can exhibit one of a rich variety of surface motility modes, e.g*.* crawling, gliding or twitching [13]. Many longer-term interactions involve biological changes within a cell, such as the formation and degradation of pili as cells crawl on surfaces [14], or the up- or down-regulation of genes associated with biofilm formation [15]. Prior to any of these biological processes, a swimming cell encountering a surface will experience forces due to the initial approach to, or collision with a surface, the details of which will probably encompass both mechanical and hydrodynamic interactions. The transfer of momentum during the interaction results in a ‘swim pressure’ exerted on the substrate [16], and after the interaction the cell body axis typically aligns with the surface. This type of effect has been shown to result in surface accumulation and curved swimming paths in a variety of bacterial and eukaryotic species, either through steric constraints or hydrodynamic interactions [17–25].

Differences between the dynamics of passive Brownian suspensions and those of active microswimmers have led to fascinating new physical insights. An example is the phenomenon of bacterial ‘rectification’, observed in fluids containing microscale asymmetric objects comparable in size to the distance between cell reorientations. At equilibrium, the density of passive Brownian particles in such environments is given by $\rho (r)∼\text{exp}\left[-\frac{U(r)}{k_{B}T}\right]$, where U(r) is an external potential. In the absence of an external potential, ρ(r) therefore constant and the particle density is uniform. In contrast, active swimmers are deflected by collisions with boundaries, and these changes in orientation can result in an inhomogeneous distribution of particles. This effect can be used to locally enrich populations of swimmers, for example in a chamber bisected by a wall composed of v-shaped obstacles, a device known as a ‘bacterial rectifier’ [26–29]. The operating principle of the bacterial rectifier has been observed in nature, where it is exploited by a carnivorous plant to trap microbial prey [30], and in medical devices to mitigate infections [31]. Understanding the physics of rectification will allow engineers to design better devices for sequestering motile microorganisms.

Hitherto, the majority of experiments on bacterial rectifiers have been conducted using the peritrichously flagellated *E. coli*. To examine the generality of rectification, we have studied the three-dimensional swimming behaviour of four bacterial species and two archaeal species, examining their surface accumulation and their behaviour as they arrive at, or leave a solid surface. We observe surface accumulation in all species, although the strength of the effect (surface-associated fraction of the population) is somewhat variable. Moreover, the track shapes as cells approach and leave the surfaces are fairly consistent, which is surprising given the variety of motility patterns among the strains that we examine. Our findings make the intriguing suggestion that the same shape of artificial rectifier may be suitable for many flagellar/archaellar arrangements.

## 2. Methods

### (a) Cell culture

The data examined in this work were created for previous studies, though the analysis here is novel. The cell culture methods in each case have been described elsewhere, but we re-iterate them briefly here. *E. coli* cells (strain HCB1, peritrichously flagellated) were grown overnight in LB media to saturation, at 30${,}^{\circ }$C and shaken at 150 r.p.m. Ten microlitres of the saturated culture was used to inoculate fresh TB media, in which the cells were grown to mid exponential phase (OD${,}_{600}∼0.3$, approx. 5 h). The cells were then washed three times into a nutrient-free motility buffer to maximize motility [32,33]. *S. enterica* serovar Typhimurium cells (strain SJW1103, peritrichously flagellated) were grown overnight in LB at $37^{\circ }$C and diluted by a factor of 1 : 200 into motility buffer [34]. *C. jejuni* cells (strain DRH212, amphitrichouosly flagellated) were grown from frozen stock on Mueller–Hinton agar plates supplemented with 10 *μ*g ml^−1^ Trimethoprim (MHT), for 20−24 h at 37${,}^{\circ }$C. The cells were washed off the surface of the plates and into fresh MH broth (media in the absence of the antibiotic), with the addition of 0.5% methylcellulose by pipetting, as previously described [35]. A strain of *Shewanella putrefaciens* that expresses only a single polar flagellum (*ΔflaA${,}_{2}$B${,}_{2}$* [36]) was grown overnight in LB, then a 10 *μ*l volume of the saturated culture was added to fresh media and grown to exponential phase (OD${,}_{600}∼0.6$) in fresh media, and placed in a shaking incubator at 30${,}^{\circ }$C and 150 r.p.m. Cells were then diluted 100-fold into fresh media for imaging. The environmental isolates of halophilic archaea used in previous work [37], HGSL (from Great Salt Lake, Utah, USA) and HXBM (from Boulby Mine, Redcar and Cleveland, UK), were grown to saturation in modified growth medium (MGM [38]) prepared without NaHCO${,}_{3}$ and NaBr, at 45${,}^{\circ }$C and 150 r.p.m. All strains were diluted into fresh media to a concentration of approximately 10${,}^{6}$ cells ml${,}^{-1}$ for imaging.

### (b) Sample preparation and imaging

Sample chambers were constructed from glass slides and coverslips using UV-curing glue [39], resulting in a sample volume measuring approximately 20 × 5 × 0.3 mm${,}^{3}$. These sample chambers were filled using a pipette, by capillary action, and sealed with petroleum jelly. The surface chemistry of the boundary is likely to be important in the general case; we controlled for this by using the same brand of glass slides and coverslips for all experiments and although a few cells were found attached to the surface, we saw no large-scale accumulation of a surface layer of static cells.

**Figure 1.**
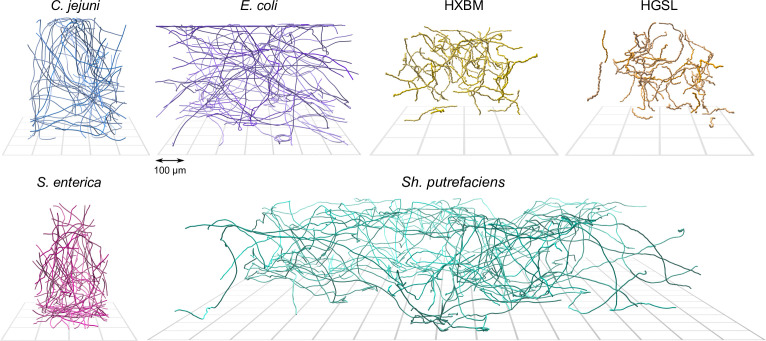
Example computer-rendered three-dimensional tracks from the four bacterial and two archaeal strains studied. The names of the species are indicated within the figure; the designations ‘HGSL’ and ‘HXBM’ refer to the two archaeal species (environmental isolates) which have not been formally described, but whole-genome sequencing in a previous study suggested that they were members of the genera *Haloarcula* (HGSL, from Great Salt Lake, USA) and *Haloferax* (HXBM, from Boulby Mine, UK). These archaeal strains swim much slower than the bacteria we examined, and consequently the effect of Brownian motion on the tracks is more pronounced, giving them a ‘crooked’ appearance. The peritrichous *E. coli* and *S. enterica* display a run-and-tumble swimming motif, while the monoflagellate *Sh. putrefaciens* shows sharper re-orientations. The other bacterial strain, *C. jejuni*, is a relatively fast swimmer that shows fewer reorientations within our sample volume. The individual panels are approximately equal in scale, and the grids beneath each set of tracks, which show the location of the sample chamber's lower surface, have a spacing of 100 *μ*m.

We tracked cells in three spatial dimensions and time using digital holographic microscopy [23,37,39,40]. Samples were placed on an inverted microscope with a bright-field objective lens (20× magnification, NA 0.50) and illuminated using a laser with a wavelength of 642 nm, and an intensity of 1.5 mW cm${,}^{2}$. The microscope was focused at a position approximately 30−50 *μ*m below the bottom surface of the sample volume. Holographic videos of 1024 × 1024, or 512 × 512 pixels were acquired using a CMOS camera at 30 Hz (HGSL, HXBM, *E. coli*), 50 Hz (*Sh. putrefaciens*, *S. enterica*) or 200 Hz (*C. jejuni*). Reconstruction was performed using Rayleigh–Sommerfeld back-propagation [41] to create a stack of images parallel to the focal plane (x,y) and spaced along the optical (z) axis. These image stacks were segmented using a method based on the Gouy phase anomaly [42], yielding the positions of the cells’ centres of mass. The volumes imaged for each species are shown in figure 1 and extend to between 400 × 400 × 500 and 1400 × 1400 × 500 *μ*m^3^.

To construct cell tracks, image coordinates were linked between frames based on their geometrical distance [39]. The uncertainty in cell location is approximately ± 1 *μ*m in the lateral directions and ± 2 *μ*m in the axial direction. To mitigate against noise in the position measurements, cell tracks were regularized using a procedure based on a piecewise cubic spline fit built into our LabVIEW analysis software [43]. This procedure examines motion in each spatial dimension ($x_{i}(t),y_{i}(t),z_{i}(t)$) and uses these to create smoothed functions (${\tilde{x}}_{i}(t),{\tilde{y}}_{i}(t),{\tilde{z}}_{i}(t)$). These smoothed functions minimize the following expression:


$$p\Sigma_{i=0}^{n-1}(x_{i}-{\tilde{x}}_{i}{)}^{2}+(1-p)\int {\left({\tilde{x}}^{\prime \prime }\right)}^{2}ds\;\;\;\text{(sim. y, z)},$$

where p is a ‘balance parameter’ allowing a trade-off between deviation from the measured points and curvature of the smoothed function, s is the arc length and n is the number of points in the trajectory. A second cubic spline interpolation scheme ensuring continuous first and second spatial derivatives was applied to fill gaps in trajectories (up to a maximum of four frames) in the cases where the detection routine failed to register a cell’s position. Fewer than 3% of frames in any trajectory were interpolated in this fashion. Tracks shorter than 1 s were discarded, except in the case of the fast-swimming *C. jejuni*, for which tracks longer than 0.5 s were retained.

## 3. Results and discussion

Figure 1 shows 50 example tracks from each species studied. The horizontal extent of each track series differs between strains because some of these data were produced for previous studies investigating different aspects of cellular motility. Nevertheless, the data showing the behaviour of cells approaching the sample chamber surface are comparable.

**Figure 2.**
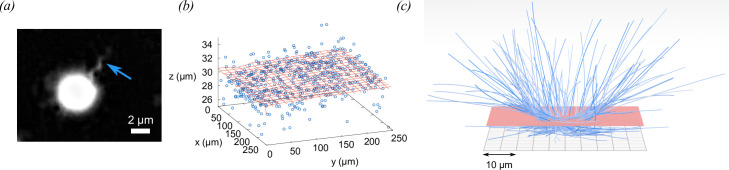
Archaeallar imaging, and identification of surfaces. (*a*) A high-intensity dark field image of a cell of archaeal strain HGSL, with the archaellar bundle indicated by a blue arrow. The flagellar bundle rotates in both directions alternately, allowing the cell to move with the bundle at the front or the back of the body. (*b*) To identify the position of the lower chamber surface, the region close to the surface was divided into ‘tiles’ in the lateral (x,y) plane. In each tile, the most frequent lowest position that cells were observed was taken to be an estimate of the lowest attainable axial coordinate. Each tile contributes one such estimate (blue points). A two-dimensional polynomial surface (order 3, red grid) was fitted through these points, and used to determine the height of the cell above the lower surface, $h_{l}$. A similar procedure was used to obtain distances from the upper surface ($h_{u}$). (*c*) A computer rendering of sections of *C. jejuni* tracks as cells approach the lower surface of the sample chamber. The red surface is located at $h_{l}=5$ *μ*m, indicating the ‘close approach’ distance threshold.

The arrangements of archaella around archaeal cells is sparsely described in the literature, although descriptions of some model organisms are available. An elegant study of the archaellar apparatus of the halophile *Halobacterium salinarum* showed steps in rotation by labelling the filaments [7]. We encountered difficulty in labelling the archaella of our strains, and so instead performed dark field imaging [44] with a specialized high-intensity LED to visualize the unlabelled filaments. An example image is shown in figure 2*a*. These cells appear to have an archaellum, or bundle of archaella, at one end of the cell which can either push or pull the cell depending on its direction of rotation (see electronic supplementary material, video). This is in good agreement with previous work that showed that these halophile strains swim with a strictly run/reverse motif [37]. Although this is surprising in the context of low-Reynolds-number hydrodynamics, where reciprocal motion does not achieve a net translation after one cycle of motion (neglecting, e.g. chemotaxis effects [45]), Brownian motion breaks symmetry and enables the archaea to explore space [46].

Hydrodynamic interactions have an increasingly large effect as cells approach boundaries. To assess these effects experimentally, it is critical to get good estimates of the cell-boundary distances, which might be confounded if the chamber surface is not flat, or not parallel to the focal plane. Although the glass slides and coverslips from which we construct the chambers are locally flat, we cannot rule out small inclinations or deformations in the chamber surfaces (especially the glass coverslips) that result in the microscope’s focal plane and the chamber surfaces being non-parallel. Furthermore, we cannot rule out any deviations from flatness incurred in the chamber construction process. Additional care was therefore taken to locate the chamber surfaces in three dimensions. Regions close to the top and bottom surfaces of the images volume were divided into 10${,}^{4}$ square tiles (100 along a side in x and y), and the closest distance of cells’ approaches to the surfaces were recorded in each tile. The relatively low magnification that we use to track cells over large distances results in an axial uncertainty in cell position of ±2 *μ*m; this uncertainty stems from noise in the imaging camera and subtle variations in the size/optical properties of the cells. A two-dimensional matrix of closest-approach values recorded in this way allows a quick inspection to determine the approximate location and any curvature of the chamber surface, and to allow the fitting bounds for the chamber surfaces to be set manually, a task that is challenging when all the tracks are plotted together. We fitted a two-dimensional polynomial surface (order 3) through the data points recovered in this way, as shown in figure 2*b*. The fitted surfaces were verified by manual examination of the calculated image stacks [41] to locate imperfections on the chamber boundaries, and are taken to be the closest that cells can approach the top and bottom of the sample chamber. We use these fits to introduce height coordinates such that a cell lying on the lower surface has a height $h_{l}=0$, or $h_{u}=0$ if it lies on the upper boundary. The distance between the upper and lower surfaces is not constant throughout the sample chambers, so the distance from each surface is noted separately for all cells, for all times. Using $h_{l},h_{u}$ calculated in this way, we define a surface-associated region where $h_{l}<5$ *μ*m or $h_{u}<5$ *μ*m. This threshold distance was chosen because it was larger than the uncertainty in the cells’ axial positions, and approximately the size of the cell body, or cell body plus flagellum in all cases. Example sections of *C. jejuni* tracks that approach the lower surface threshold are shown in figure 2*c*.

**Figure 3.**
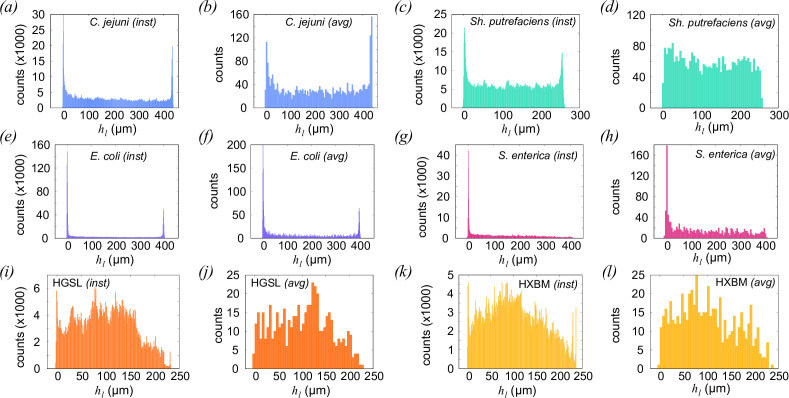
Instantaneous and track-averaged distances of cells from lower boundaries, with species and calculation method indicated in the panels. To construct the ‘instantaneous’ distribution of positions, each cell contributes one count to the histogram at each time point. The ‘average’ histograms are constructed by determining the average value of $h_{l}$ for each track, with each track contributing one count to the histograms. Numbers of data points used to compose the instantaneous (average) distributions: *C. jejuni* 1.40 $\times 10^{6}$ (2998); *E. coli* 1.51 $\times 10^{6}$ (2882); HGSL 7.48 $\times 10^{7}$ (523); HXBM 6.34 $\times 10^{7}$ (594); *Sh. putrefaciens* 1.65$\times 10^{6}$ (3000); *S. enterica* 5.75 $\times 10^{5}$ (1374).

We investigated surface accumulation in our six strains by finding the instantaneous and track-averaged positions of cells in our sample chambers, as shown in figure 3. The distribution of cell densities is plotted as a function of height from the lower surface, $h_{l}$. The results found when plotting as a function of $h_{u}$ are almost mirrored, but otherwise similar. All strains show some degree of accumulation at surfaces, but this is most pronounced in *E. coli* and *S. enterica*, our two peritrichously flagellated strains. All the samples show cells preferentially accumulating at lower $h_{l}$, presumably due to the effects of gravitational sedimentation. This will result in a density profile ρ(z)∼exp(−κz) with a decay length $\kappa^{-1}∼1$ mm for typical bacterial cells [47], substantially larger than the height of our sample chambers. The polar- and amphitrichously flagellated strains (*Sh. putrefaciens* and *C. jejuni*, respectively) appear to spend a larger proportion of their time in the bulk fluid within the sample chamber. Here, the results from *Sh. putrefaciens* are outliers: although the cells make occasional deviations close to the chamber walls (distribution of instantaneous positions (figure 3*c*)), the data indicate that this is the exception, and we see no enrichment of the wall-adjacent population in the average position data (figure 3*d*). These data emphasize the need to consider species on a case-by-case basis; theory and numerical work based on a ‘microswimmer’ model must be informed or constrained by experimental observations. The results from archaeal strains HGSL and HXBM show some evidence of a peak at $h_{l}=0$ *μ*m, particularly in the instantaneous position distributions, and a modest depletion of cells in the upper third of the chamber in both species. This suggests some degree of sedimentation; by way of comparison, neutrally buoyant Brownian particles would be expected to have a uniform distribution throughout the sample.

**Figure 4.**
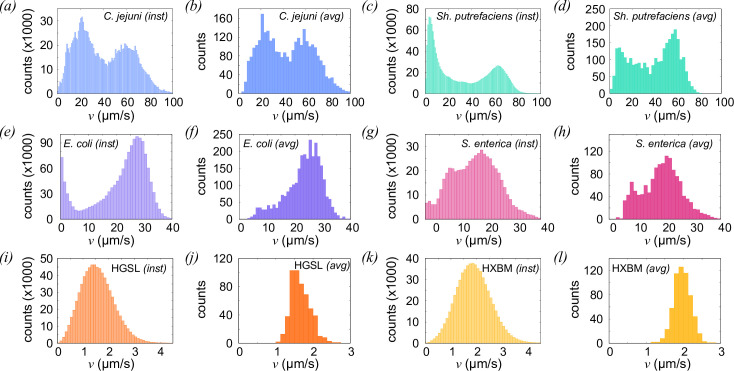
Distributions of instantaneous (‘inst') and average (‘avg') swimming speeds of microbial cells. The numbers of data points in each case are the same as for figure 3. The average speeds are: *C. jejuni* 43.48±.01 *μ* ms${,}^{-1}$; *E. coli* 23.14±.01 *μ* ms${,}^{-1}$; HGSL 1.667±0.004 *μ* ms${,}^{-1}$; HXBM 1.968±0.005 *μ* ms${,}^{-1}$; *Sh. putrefaciens* 50.14±0.03 *μ* ms${,}^{-1}$; *S. enterica* 18.77±0.01 *μ* ms${,}^{-1}$. The quoted uncertainties are the s.e. on the mean.

Figure 4 shows the instantaneous and track-averaged speeds of all six microbial strains. Most track-averaged distributions are approximately single-peaked with the exceptions of *C. jejuni* and *Sh. putrefaciens*, which have bimodal distribution of speed in both instantaneous and averaged distributions. Individual cells of *Sh. putrefaciens* are observed to transition between fast and slow populations, but those of *C. jejuni* do not appear to do so on the time scale of our observations. Our qualitative observation is that while *E. coli* cells’ trajectories are momentarily interrupted by collisions with the surface, this phenotype is seen less clearly with the other peritrichously flagellated species, *S. enterica* (data not shown). The same batch of samples chambers was used in both cases, and the culture media was also similar, ruling out a trivial explanation for these differences. We speculate that the reason for the difference here is that the molecular composition of the flagellar filaments differs between these two species, especially in the outer part of the flagellar subunit proteins (flagellins). Flagellins are triggers for the immune response in humans and other animals and are therefore subject to strong selective pressure. Variations in the structure of the external regions of flagellin subunits between different species and strains are well established [48], so slight differences in their interaction with the glass substrate are not unexpected. The speed distributions for the archaeal species show a much slower average swimming speed, commensurate with previous studies [37,49]. Brownian motion significantly affects the interpretation of the swimming motion of these halophilic archaea, and smoothing is required to regularize the trajectories and extract more reliable estimates of swimming speed. The regularization process is responsible for the smooth, approximately Gaussian distribution of speeds observed, though the average speed we measure is consistent with the cells’ net displacement over several seconds.

**Figure 5.**
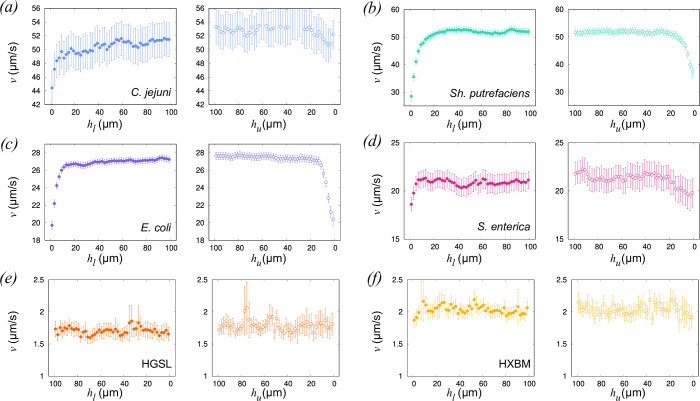
The instantaneous speeds of cells approaching and leaving the region adjacent to a no-slip boundary. The error bars represent 95% CI, and the numbers of cells in each set are the same as those in figure 3.

Figure 5 shows the speeds of the cells as they approach or leave a surface. The data have been placed in bins of width $\Delta h_{u,l}=$2 *μ*m, and the data points show the mean speed in each bin. The error bars reflect 95% confidence intervals on the mean within one bin. In all bacterial species, speed noticeably decreases close to the surface. While this can be accounted for at the closest distances by collisions between the cells and the wall, the region in which speed is reduced extends to $h_{l,u}\approx 15\;$*μ*m in each bacterial case. The details of the interactions between cell and surface are likely to be complicated and nuanced. The fluid friction experienced by a body increases when it moves close to a no-slip boundary. Corresponding corrections to the dynamic viscosity are known analytically for simple objects such as spheres (e.g*.* Faxén’s correction [50]), but the complicated geometry of cell bodies and flagella/archaella makes similar calculations for bacteria/archaea challenging. Other effects that might affect swimming speed include the apparent suppression of cell body wobbling that results from the proximity to a surface in colloidal or polymer suspensions [51]. This has been posited as the cause of anomalies such as an apparent increase in swimming speed in viscoelastic suspensions [52,53]. We find that cells simply slow down in the region next to the boundary. If cells become intermittently stuck to the interface, we might expect to see the speed distribution broaden for cells close to the surface, increasing the uncertainty in the mean speed as $h_{u,l}\to 0$. We see no such increase in the standard error and conclude that while surface collisions do occur, the decrease in average swimming speed can be attributed mainly to an increase in friction. The decrease in cell speed looks weaker in the case of the archaeal cells (especially HGSL, figure 5*e*), but both the scatter in the data and the fractional uncertainty (i.e. uncertainty divided by mean value) are larger for these strains. In all the strains, we see some amount of slowing close to the boundary, but it is difficult to determine whether this is because cells slow down near the boundary, or because the cells that swim slowly accumulate at the boundary. There are two reasons for this ambiguity in our experiments. First, we have limited ability to track cells over very long trajectories allowing for repeated approaches to the surface (cell speed fluctuates somewhat while they are swimming). Second, and more fundamentally, if a cell remains in the surface region, it is impossible to tell how fast it would have swum if it were in the bulk.

The effect of a nearby interface will contribute to curvature in the tracks, most intuitively because the cells must realign their swimming direction when they encounter a surface. To obtain the curvature, we employ the Frenet–Serret convention on track data expressed as a series of points in space r(s). Tangent vectors are obtained by taking the spatial derivative of r(s), and the curvature results from the second spatial derivative:


(3.1) $$\begin{array}{lcr} & \hat{\text{t}}(s)={\text{r}}^{\prime }(s)/|{\text{r}}^{\prime }(s)|\;\;\text{and}\;\;\;\kappa =|{\hat{\text{t}}}^{\prime }(s)|. & \end{array}$$

For our discrete data, we evaluated spatial derivatives by dividing the difference between subsequent coordinates by their scalar Euclidian separation.

**Figure 6.**
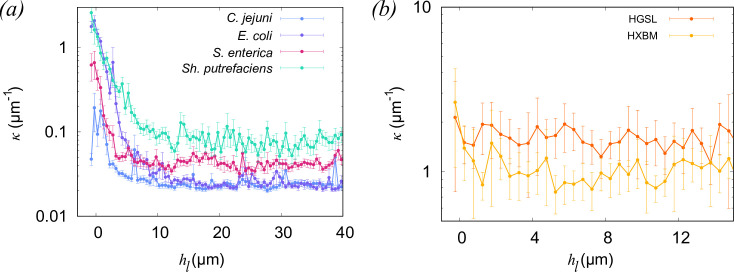
Track curvature as a function of distance from the boundary. The results are derived from instantaneous data, and displayed on a log-scale on the vertical axis. (*a*) The results for bacterial samples, and (*b*) results for archaeal strains. The error bars represent approximately 95% CI. The numbers of cells in each set are the same as those in figure 3.

Figure 6 shows the curvature of tracks obtained by this method. The bacterial cells (figure 6*a*) show significant curvature as $h_{l}\to 0$, with *Sh. putrefaciens* experiencing the greatest effect. The slow-swimming archaeal strains (Figure 6*b*) show essentially no variation in curvature among the cells in close proximity to the surface; they may exhibit a slight increase in track curvature at the very closest distances, but we note that the uncertainties at $h_{l}\approx 0$ are large. Calculated in this way, curvature must be greater than zero, which explains why the curvature is finite in the region $h_{l}\geq 20\;$ *μ*m. A potentially fruitful area for future research would be examining how in-plane and out-of-plane curvature might be distinguished, to give more detailed insight into the cell-boundary hydrodynamics interactions.

**Figure 7.**
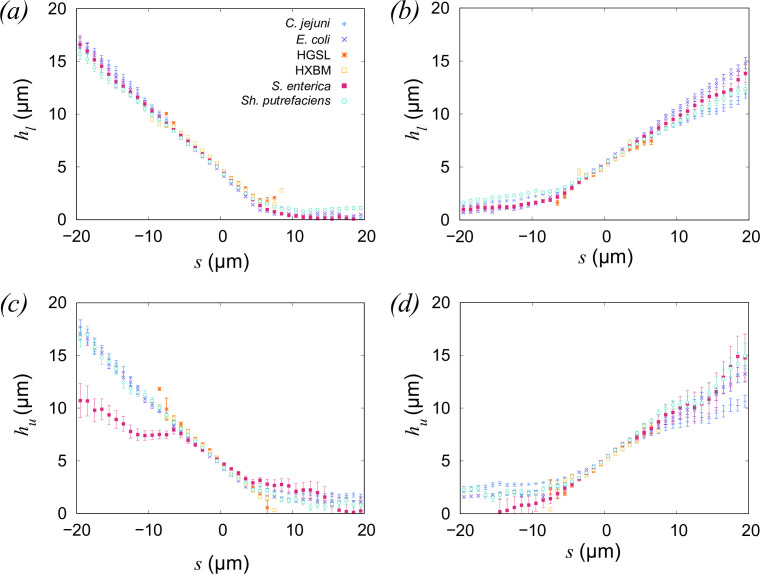
Trajectories of cells approaching and leaving the regions adjacent to the chamber surfaces. The distances of the cells from the lower surface ($h_{l}$) and upper surface ($h_{u}$) are plotted as a function of arc length (s). The tracks have been arranged so that s=0 is taken to be the point at which the trajectory crosses a plane 5 *μ*m from the relevant surface. The error bars represent approximately 95% CI.

Next, we examined the shape of tracks on approach to, and leaving a surface. Our strains exhibit a 30-fold difference in swimming speed between the fastest and slowest strains (*Sh. putrefaciens* and HGSL, respectively), so to provide a better comparison between the behaviour of the cells as they approach the surface, we examine the shape of the tracks, independent of time. We extracted 40 *μ*m segments of tracks (20 *μ*m for the archaeal strains) that cross a threshold of 5 *μ*m from either the upper or lower surface, when arriving or leaving this region. The total numbers of track segments are listed in table 1. The distance from the upper and lower surface ($h_{u},h_{l}$, respectively) are plotted as a function of s, the distance along the track contour, in Figure 7. Values of s are referenced to the position at which they cross the surface threshold $h_{u,l}=5$ *μ*m, which we define as s=0 *μ*m. Cells can approach a surface from any angle, and we observe that the average angle of approach is approximately constant between the various species. This is to be expected, assuming that the cells interact only weakly with surfaces at a distance of tens of micrometers, and that swimming is generally isotropic (figure 7*a*,*c*). The angle between approaching cell trajectories and the surface is approximately 35${,}^{\circ }$, which is slightly steeper than the angle expected from strictly ballistic motion (approximately 30${,}^{\circ }$, assuming that the angle of approach can lie anywhere on a unit hemisphere). We also note that this simple approximation neglects the effects of curvature, and trajectories at glancing incidence which will lie entirely within the 5 *μ*m surface threshold do not appear in our analysis.

**Table 1.** Numbers of tracks segments from each strain approaching and leaving the top and bottom surfaces of our sample chambers.

species	lower surface		upper surface	
	approaching	leaving	approaching	leaving
*C. jejuni*	131	120	55	41
*E. coli*	343	270	187	145
HGSL	30	9	10	14
HXBM	42	7	14	7
*S. enterica*	133	116	13	11
*Sh. putrefaciens*	375	372	369	379

Figure 7*b*,*d* shows that there is more variation in the angles with which the cells leave the surface. After leaving the region close to the boundary, the tracks of the amphitrichous *C. jejuni* are more closely aligned with the surface than those of the other strains. This may reflect the relative difficulty of achieving a successful ‘escape’ from surface association if the cells are limited only to reversing direction as opposed to executing the randomly oriented tumbles seen in *E. coli* or *S. enterica*, for example. The data for *S. enterica* approaching a surface in figure 7*c* appear to deviate from the mean path experienced by the other bacterial species, but we note that the number of cells is low (table 1), consistent with the data in figure 3*g*,*h*. The rates at which cells leave the upper and lower boundaries are remarkably similar, suggesting that the physics that both experience is dominated by the hydrodynamic and steric effects of the boundary, and that gravity plays a relatively minor role for these cells. The results for the archaeal strains are broadly similar to those in previous examinations of the data, in that the surface seems to impinge minimally on the swimming behaviour of the cells. This we attribute to the slow and erratic swimming of the halophilic archaea, for which translational Brownian motion plays a larger role than it does for their faster-swimming bacterial counterparts. Understanding the advantage of slow swimming is an outstanding challenge in the study of bacterial, archaeal and other microswimmers. Particularly in the case of halophilic archaea, swimming speeds in the range 1−5 *μ*m s${,}^{-1}$ seem universal, and have been observed both in lab-grown samples and those taken directly from the natural environment [37,44,54]. Maintaining and reproducing the motility apparatus requires energy, and operating it consumes ATP [55]. Given that many archaea are sparsely distributed in nutrient-poor environments, we infer that there must be a significant advantage in swimming, even slowly.

Finally, the ability of rectification devices to capture and modify the swimming behaviour of cells is intrinsically linked to the amount of time that a cell remains in the surface-adjacent region. To evaluate this quantity for our strains, we extracted sections of swimming trajectories where cells approach the surface ($h_{l,u}\leq 5$ *μ*m), and then leave again. For these sections, we measure the duration of the surface-associated period (‘dwell time’, $\tau_{s}$) and the end-to-end distance travelled ($r_{E}$) while the cell is in the surface-associated region. The results are listed as mean values with uncertainties representing the s.d. in table 2.

**Table 2.** The surface dwell time ($\tau_{s}$) and the distance travelled while the cell is in the surface-adjacent region ($r_{E}$). The numbers represent the mean value, and the uncertainty reflects the s.d.; N is the number of close approaches to the surface recorded in each dataset.

species	lower surface			upper surface		
	$\tau_{s}$	$r_{E}$	N	$\tau_{s}$	$r_{E}$	N
	(s)	( *μ*m)		(s)	(*μ*m)	
*C. jejuni*	1.38 ± 0.93	45.97 ± 26.60	107	0.77 ± 0.48	33.25 ± 15.43	43
*E. coli*	6.88 ± 7.81	59.59 ± 67.56	366	4.95 ± 5.99	51.53 ± 68.57	385
HGSL	17.10 ± 26.27	11.73 ± 17.69	17	3.82 ± 6.03	3.48 ± 5.41	15
HXBM	23.19 ± 33.59	28.96 ± 45.77	3	9.56 ± 14.14	7.29 ± 8.34	10
*S. enterica*	3.84 ± 3.67	47.09 ± 44.37	85	1.69 ± 1.55	12.49 ± 9.39	6
*Sh. putrefaciens*	1.45 ± 1.67	29.85 ± 23.72	875	1.24 ± 1.46	26.71 ± 22.22	741

Consistent with the results in figure 3, the amount of data available for the archaeal strains is an order of magnitude lower than that for bacterial strains, because the archaea show weaker surface accumulation. Intriguingly, there are noticeable differences in $\tau_{s}$ between bacterial strains, with the amphitrichously flagellated *C. jejuni* and polar flagellated *Sh. putrefaciens* able to escape surfaces more quickly. The distances travelled within the surface regions ($r_{E}$) are much more closely distributed, however. The bacteria with the smallest and largest $r_{E}$ (*Sh. putrefaciens* and *E. coli*, respectively) differ by approximately a factor of two, but the distributions are broad, and all bacterial samples have mean values of $r_{E}$ that lie within one s.d. of the others, both on the bottom and top surfaces of the sample chamber (notwithstanding a very small number of cells at the top of the chamber for *S. enterica*). This lends support to the idea that the same rectifier could trap multiple species of bacteria, as the geometry of the tracks, as opposed to their time-dependent interaction with cells, governs the rectification efficiency. Further work in this area might look at examples where individual cells can be tracked for longer times [56], as this could show evidence for fluctuations in the internal state of cells, for example longer-term changes in speed, reversal frequency and so on between species.

## 4. Conclusion

Interactions between microbes and surfaces are common in nature, and essential to a broad range of processes from infection to biofilm formation. We used three-dimensional cell tracking to study the near-surface behaviour of swimming cells with a variety of swimming patterns. We observe that the surface-accumulation effect noted by others is present, at least to some extent, in all strains. Some are resident for significantly longer in the surface-adjacent region than others. For example, direct collisions lead to *E. coli* cells having a large population of slow-swimming cells close to the solid interface, whereas *Sh. putrefaciens* only makes occasional detours close to the surface, with the majority of cells remaining in the bulk, on average. The motility patterns we investigated included run-and-tumble (*E. coli*, *S. enterica*), run-reverse (HGSL, HXBM), run–reverse–flick (*Sh. putrefaciens*) and a mixture of the two latter modes (*C. jejuni* in methylcellulose). Despite these differences, we observe that on average, the geometry of their swimming paths on departure from a solid interface are similar. This somewhat unexpected result simplifies the design considerations for devices intended to ‘rectify’ bacterial swimming to enrich or sequester motile populations. If different species of motile bacteria leave a surface in more or less the same fashion, parameters such as run length (the spatial distance between re-orientations) will dominate the rectification dynamics for all species. By reducing the parameter space of possible designs in this way, a general device for capturing a range of different species could feasibly be produced.

## Data Availability

The data underlying our results can be found in our institutional repository [57]. Supplementary material is available online [58].
